# Single-copy gene based 50 K SNP chip for genetic studies and molecular breeding in rice

**DOI:** 10.1038/srep11600

**Published:** 2015-06-26

**Authors:** Nisha Singh, Pawan Kumar Jayaswal, Kabita Panda, Paritra Mandal, Vinod Kumar, Balwant Singh, Shefali Mishra, Yashi Singh, Renu Singh, Vandna Rai, Anita Gupta, Tilak Raj Sharma, Nagendra Kumar Singh

**Affiliations:** 1 National Research Centre on Plant Biotechnology, Indian Agricultural Research Institute, New Delhi 110012, India; 2 Rayat and Bahra Institute of Engineering and Bio-Technology, Mohali, Punjab Technical University, Jalandhar 140104, India

## Abstract

Single nucleotide polymorphism (SNP) is the most abundant DNA sequence variation present in plant genomes. Here, we report the design and validation of a unique genic-SNP genotyping chip for genetic and evolutionary studies as well as molecular breeding applications in rice. The chip incorporates 50,051 SNPs from 18,980 different genes spanning 12 rice chromosomes, including 3,710 single-copy (SC) genes conserved between wheat and rice, 14,959 SC genes unique to rice, 194 agronomically important cloned rice genes and 117 multi-copy rice genes. Assays with this chip showed high success rate and reproducibility because of the SC gene based array with no sequence redundancy and cross-hybridisation problems. The usefulness of the chip in genetic diversity and phylogenetic studies of cultivated and wild rice germplasm was demonstrated. Furthermore, its efficacy was validated for analysing background recovery in improved mega rice varieties with submergence tolerance developed through marker-assisted backcross breeding.

Rice, one of the most important food crops, is a staple diet for over half of the human population. Rice has played a central role in human nutrition and civilisation for at least past 10,000 years^1^. Next-generation sequencing (NGS) technologies produce huge volumes of nucleotide sequence data in single runs, providing inexpensive genome-wide sequence reads that have led to the discovery of several SNPs with a density of approximately three to four SNPs per kilo-base pairs (kbp) in the rice genome^2^. Because of their high abundance, bi-allelic co-dominant inheritance, and sequence tagged nature, SNPs have the potential to provide the basis for superior and highly informative genotyping assays. After the publication of high quality reference genome sequence data of rice^3^, several re-sequencing projects have contributed considerably to the information on the genome structure and genetic diversity of rice^4^,^5^,^6^,^7^,^8^. SNP genotyping platforms can be grouped broadly into two categories: (i) low-multiplexing with high sample throughput for population structure studies, foreground selection, fine mapping, diagnostics, and seed quality control and (ii) high-multiplexing with low sample throughput for association mapping, background selection, genomic selection, and evolutionary studies.

Genotyping by sequencing (GBS) and high-density array-based SNP detection are the two major high-multiplexing SNP genotyping platforms. Although GBS is highly efficient and cost effective, its experimental operation involving extensive data analysis is beyond the capabilities of an average rice breeding group^9^. In contrast, high-density arrays can be used for rapidly genotyping several common SNPs across samples with relatively easy data analysis, although these assays are expensive. A high-resolution 44 K Affymetrix array and a 50 K Infinium array (RiceSNP50) have been developed for rice SNP genotyping^10^,^11^,^12^,^13^. In addition, efficient high-density SNP arrays have been developed for other crop plants, including maize and sunflower^14^,^15^, as well as domestic animal species, including cattle (BovineSNP50^16^,^17^) and pig (PorcineSNP60^18^). However, most of these arrays include random genome-wide SNPs, and none is based specifically on single-copy (SC) genes.

Several SC conserved orthologous genes (COSII) were first described in the euasterid clade of flowering plants and were found useful for comparative, evolutionary, and systematics studies^19^. Comparative analysis of 7,241 SC rice gene homologs in wheat provided new insights into the conservation of synteny and evolutionary changes caused by the duplication, divergence, and transposition of genes^20^. A comprehensive analysis of unique (SC) genes in *Arabidopsis* and rice genomes revealed two types of SC genes: (i) newly evolved species-specific SC genes and (ii) ancient SC genes conserved between the two species that separated from a common ancestor more than 725 million years ago^21^. Additional studies identified 959 shared SC genes among *Arabidopsis*, *Populus*, *Vitis*, and *Oryza*, most of which also seem to be conserved in other plant species^22^. Furthermore, several conserved SC genes were identified in a study of 40 vertebrates, 23 arthropods, and 32 fungal genomes^23^. Studies investigating several flowering plants have shown the existence of ‘duplication-resistant’ SC genes that have retained their SC status despite multiple genome-wide duplications^24^,^25^. Key features of conserved SC genes common to multiple species are their ancient origin, high level of expression in general and in more tissues compared with non-SC genes, and low level of sequence divergence^21^,^24^. These genes are often involved in essential housekeeping functions or they lack proper functional annotation^20^,^24^. Thus, a SC gene based SNP array may prove an efficient tool for studying evolutionary relationships among different *Oryza* species and mapping of genes and quantitative trait loci (QTLs) for complex agronomic traits in cultivated rice. Furthermore, this array may be useful for characterising wild rice germplasm and providing new insights into rice domestication. We here describe the design and validation of a unique SC gene based 50 K rice SNP chip (OsSNPnks) for genetic studies and molecular breeding applications in rice.

## Results

### Design and validation of SC gene based 50 K rice SNP chip

**Figure 1:**
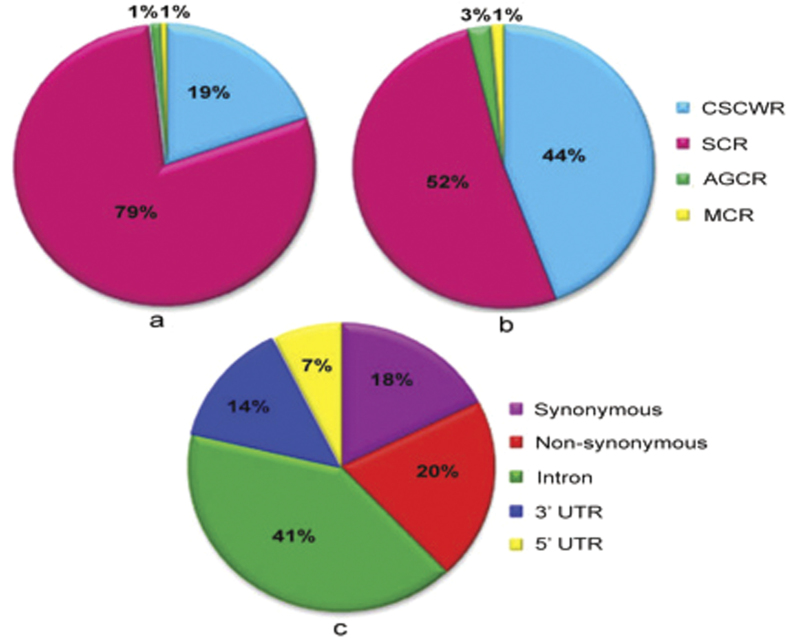
Distribution of different kinds of rice genes and number of SNPs in the Affymetrix 50 K rice SNP chip (OsSNPnks): (**a**) Number of genes in different categories; (**b**) Number of SNPs in different categories of genes; (**c**) Number of SNPs in different regions of the genes.

The custom-designed Affymetrix rice SNP genotyping chip ‘OsSNPnks’ includes 50,051 high-quality non-redundant SNPs, mostly representing SC genes from the entire 12 rice chromosomes. Four categories of rice genes used for the chip design were, conserved SC genes between wheat and rice (CSCWR), unique SC rice genes (SCR), agronomically important cloned rice genes (AGCR), and multi-copy rice genes (MCR), of which the highest proportion of genes (79%) was from the SCR category, followed by 19% from the CSCWR category (Fig. 1a). In total, 48,217 (96%) SNPs on the chip were from SC genes, and the remaining 4% SNPs, including 1,216 (3%) from AGCR and 618 (1%) from MCR genes, were included for molecular breeding applications and MCR used as control (Fig. 1b). This rice chip had 38% SNPs from exons, including 8,798 (18%) synonymous and 10,148 (20%) non-synonymous types with the potential to affect gene function, 41% SNPs from introns, and 21% SNPs from 5′ and 3′ UTR regions (Fig. 1c). The chip has a high-density genome-wide coverage with an average distance of 0.745 kbp between adjacent SNPs (Supplementary Figure 1). The AGCR genes had a high number of 6.26 SNPs per gene (1.21 SNP/kbp) perhaps due to more extensive re-sequencing studies performed on these genes. Of the two categories of SC genes, the CSCWR genes showed an over three-fold higher number of 6 SNPs per gene (1.25 SNP/kbp) than the SCR genes that showed only 1.73 SNPs per gene (0.85 SNP/kbp). The MCR genes showed a medium number of 5 SNPs per gene (1.07 SNP/kbp). However, in the chip, 26,001 (~52%) of all SNPs were from the SCR category because of the much larger number of SCR genes (Table 1). The higher average number of SNPs in the CSCWR genes than in the SCR genes is attributed to both the larger average size (4,782 bp for CSCWR vs 2,036 bp for SCR) and the ancient origin of the CSCWR genes, allowing the accumulation of more SNPs^21^,^25^. The complete array information of the 50 K rice SNP chip is provided in Supplementary Table 1.

*In silico* validation of the assay involved preliminary screening of the designed array file for each selected SNP, including their p-convert values generated using Affymetrix power tool (APT) AxiomGTv1 algorithm to ensure a high-quality final array^26^. Both forward and reverse probes of each SNP were assigned with p-convert values, derived from a random forest model to predict the probability of SNP conversion on the array. The model considers factors including the probe sequence, binding energy, and expected degree of non-specific hybridisation to multiple genomic regions. SNP probes with high p-convert values are expected to convert on the SNP array with a high probability. Potential probes were designed for each SNP in both the forward and reverse direction, each of which was designated as ‘recommended’, ‘neutral’, or ‘not recommended’ based on p-convert values through which the SNP data sets were easily filtered. Thus, SNP probes were designed by screening 55,100 SNP loci, of which an extremely high proportion of 50,051 loci (90.8%) showed high-quality scores with p-convert values of >0.30, and the vast majority of them having p-convert values of >0.6, which were successfully synthesised on the array chip. We experimentally validated the chip by genotyping a set of 192 diverse rice genotypes, including cultivated and wild rice germplasm by using an integrated Affymetrix GeneTitan^®^ platform to scrutinise the SNP array performance. All the samples passed the development quality check (DQC) with a high cut-off value of >0.92, and the maximum DQC value was 0.99. Data generated using APT and genotyping console (GTC) software were analysed. Except for one sample, all the other samples possessed high genotyping call rates of >95.5%, with an average call rate of 99.9%. Thus, the data showed high sample success and genotyping call rates. In all the genotypes, 45,611 (91.1%) SNPs had call rates of >95.5%. Among the existing Affymetrix SNP chips, the present custom-designed chip has one of the highest sample success rates of 99.4%, with no possibility of cross-hybridisation in the rice genome owing to its SC gene based array design (Supplementary Table2). Because the genotyping data quality was excellent, with an average SNP call rate of 99.9%, genotyping data imputation for haplotype analysis was avoided. Furthermore, chip reproducibility was examined using two samples in duplicate and one sample in triplicate, which showed 99.9% array reproducibility.

Call rates were compared among the four categories of genes, CSCWR and SCR genes had the highest 91.4% and 91.0% SNPs with call rates of >95.5%, respectively. AGCR genes also had a high 90.8% SNPs with call rates of >95.5%, but MCR genes showed the lowest 86.8% SNPs with call rates of >95.5%. Thus, MCR gene based SNPs used as control showed approximately 5% lower SNP call rates than SC gene based SNPs. These findings demonstrate the advantage of the SC genes based array design in SNP call rates and array reproducibility.

### Applications of the SC gene based 50 K rice SNP chip

#### Haplotype-based genetic diversity and phylogenetic analysis

**Figure 2:**
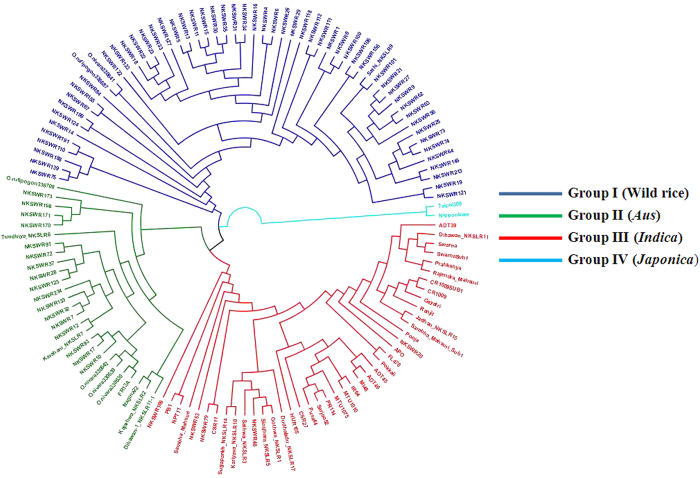
SNP haplotype-based neighbour-joining phylogenetic tree of 128 diverse rice genotypes, including wild rice, land races, improved cultivars and *Sub1* backcross derivatives of mega rice varieties. Four major distinct groups are: wild rice with ‘*sathi’* (blue), wild rice with *Aus* rice cultivars (green), wild rice with *Indica* rice cultivars (red) and *Japonica* rice cultivars (cyan).

Haplotype-based phylogenetic analysis unravels the origin of mutations and provides clues to the evolution of genetic diversity in populations. The SC gene based 50 K rice SNP chip was used to study the genetic diversity and phylogenetic relationship among 128 diverse rice germplasm, including wild rice accessions, traditional rice varieties, and genetically improved rice cultivars. Of the total 192 samples used for chip validation, 64 samples were excluded from this analysis because one sample had a low call rate, four samples were in replicates, and 59 samples were breeding lines and bulk samples (Supplementary Table 3). Haplotype based analysis separated the 128 genotypes into four major groups (Fig. 2, Supplementary Table 3). Group I comprised entirely of wild rice accessions including both *O. rufipogon* and *O. nivara*. This group represented the eastern states of India including Uttar Pradesh, Bihar, West Bengal, and Odisha. Only exception in this group was a unique extra-early maturing rice landrace Sathi_NKSLR-9 [matures in 60 (*sath*) days; therefore, named *Sathi*], which was clustered with Group I along with wild rice accessions collected from the Indo-Gangetic Plain. Group II comprised wild rice accessions along with rice landraces, including well-known *Aus* varieties, namely Nagina 22 and FR 13A, which seem to have evolved from *O. nivara*. Wild rice collected from widespread geographical regions of India were represented in this group, including those collected from Himachal Pradesh, Uttar Pradesh, Bihar, Bengal, Gujarat, and Maharashtra. Group III comprised mainly of well-known *indica* rice varieties, namely ADT 39, CR 1009, IR 64, MTU 1010, Pokkali, Pusa 44, Ranjit, Swarna, and their *Sub1* derivatives, along with five wild rice accessions (*O. nivara*), of which four accessions were from Uttar Pradesh and one accession was from Himachal Pradesh. Group IV included two accessions of *japonica* rice cultivars Nipponbare and Taipei 309 and no clustering of wild rice accessions.

### *Background recovery analysis in backcross-derived rice varieties*

**Figure 3:**
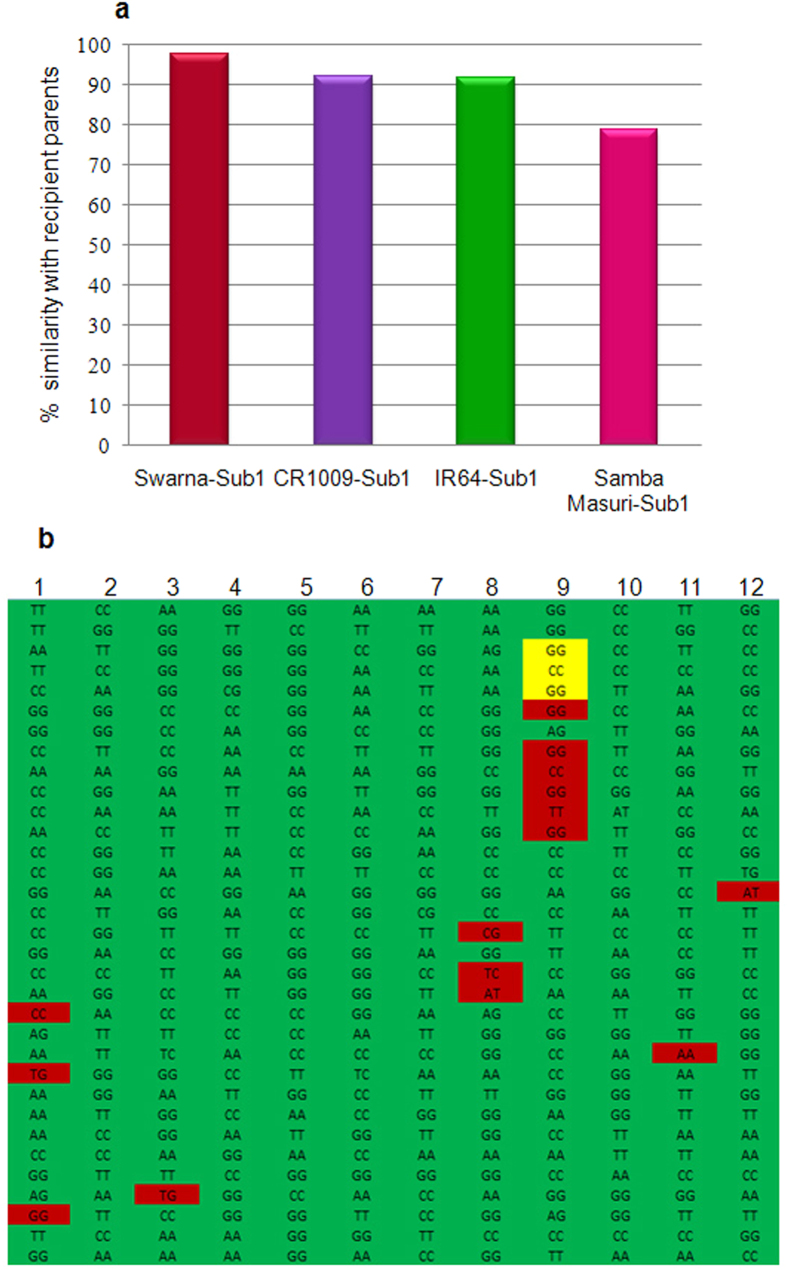
Background recovery analysis in Sub1 derivatives of four mega rice varieties: (**a**) Percentage similarity between recipient variety and its backcross derived submergence tolerant version (**b**) Snapshot of chromosome wise (1-12) similarity between Swarna and Swarna-Sub1, alleles of donor parent are indicated in red color, while *Sub1B* gene is highlighted in yellow color on chromosome 9.

The 50 K rice SNP chip was used to analyse the percentage similarity of four submergence tolerant mega rice varieties, namely Swarna-Sub1, CR1009-Sub1, IR64-Sub1, and Samba Mahsuri-Sub1, with their respective recipient parents. These rice varieties were developed by introgression of *Sub1A* gene located on the rice chromosome 9 through marker-assisted backcross breeding^27^. Backcross-derived lines with maximum recipient genome recovery (background selection) in these lines were identified using low-density SSR markers and phenotypic selection. The overall background genomes of Swarna-Sub1, CR1009-Sub1, IR64-Sub1, and Samba Mahsuri-Sub1 estimated using the 50 K rice SNP chip were 97.7%, 91.9%, 91.7%, and 78.7%, respectively. As expected, because of linkage drag, background recovery of chromosome 9, bearing the target *Sub1A* gene, was slightly lower at 94.0%, 91.5%, 86.03%, and 78.5%, respectively (Supplementary Table 4). High density background analysis by using the 50 K rice SNP chip clearly showed that a significant proportion of the residual donor background genome was present in all the four varieties spread across all the 12 rice chromosomes, with the highest background recovery in Swarna-Sub1 (Fig. 3a, b).

The available 194 AGCR gene based SNPs in the 50 K rice SNP chip allowed background analysis in terms of haplotype recovery for these agronomically important genes in the submergence tolerant derivatives of these four mega rice varieties (Supplementary Table 5). The percentage recovery of the recipient variety haplotypes of the 194 AGCR genes that influenced agronomically important traits was as follows: Swarna-Sub1 (94.9%), CR1009-Sub1 (87.2%), IR64-Sub1 (85.6%), and Samba Mahsuri-Sub1 (52.6%). Phenotypic evaluation of submergence tolerance confirmed that all the four Sub1 lines exhibited significantly greater tolerance to complete submergence than their original recipient parents, with no obvious adverse effect on the agronomic performance, including yield and grain quality, under regular growth conditions^27^,^28^,^29^. However, to date, Swarna-Sub1 is reported to have found most wide spread acceptance by the farmers in the flash flood-prone areas. The recovery of recipient parent alleles of AGCR genes was the maximum in Swarna-Sub1, with only 10 AGCR genes (5.1%) having donor type alleles (Supplementary Table 6). Most of the non-recovered genes were associated with various kinds of stress tolerance, and therefore would not have considerable effect on the agronomic performance under normal cropping conditions.

## Discussion

DNA markers are currently used widely for analysis of genetic diversity, evolutionary studies, association mapping as well as diagnostics, fingerprinting and breeding applications. Among various types of DNA markers, SNPs are the most abundant and robust, feasible for automated high-throughput genotyping, and available for multiple assay options using different technology platforms to meet the demand for genetic studies and molecular breeding in crop plants^30^,^31^,^32^,^33^. Conventional plant breeding is based on rigorous phenotypic selection and depends on breeder’s experience, hence it takes longer time to develop a new improved variety as complex quantitative traits are very difficult to select due to low heritability. SNP chip arrays allow genotyping of thousands of markers in a short time for association studies to identify DNA markers linked to these complex traits. Recently, there is strong advocacy for GBS using NGS platforms to save cost per data point, but high informatics cost and paucity of commonly genotyped loci across samples are the real twin challenges hindering the adoption of GBS^9^. The generation and analysis of SNP genotyping array data are easier and cost-effective. For fast track development of superior rice varieties, there is need to map and tag agronomically important genes and understand the basis of allelic variation at these loci. A major challenge is to identify and validate sets of informative genome-wide SNP loci from large sequence data sets that will function effectively as SNP markers.

The first step in the design of the present chip was filtering the large volume of rice genome data for ensuring that the specific SNP variants represented SC genes, which had no nearby variant that might interfere with the array design. The advantage of having a large pool of available SNPs in rice was that a comprehensive subset of SNPs belonging to the SC genes was selected for the chip design. The 50 K rice SNP chip (OsSNPnks) designed here by using the Affymetrix Axiom® myDesign Custom Array tool is highly useful for genetic studies and molecular breeding applications in rice. The novelty of the chip is that it contains 48,217 SC gene based non-redundant high-quality SNP probes in 2–4 replicates. It has a high average sample success rate (99.4%), SNP call rate (99.9%), and assay reproducibility (99.9%). The extremely high call rates and assay reproducibility were attributed to the SC gene based SNP array design with no sequence redundancy and cross-hybridisation problems, as clearly demonstrated by lower call rates of SNPs in the MCR genes used as controls. Although the Affymetrix Axiom® my Design Custom Array considers gene duplication and homology in the SNP flanking sequences, the quality of individual SNP array is reflected in the average and cut-off, DCQ, and p-convert values, which were much higher for the SC genes than for the MCR genes. Furthermore, the use of SC genes led to a high array design success rate of 90.8%. The CSCWR genes had an over three-fold higher number of SNPs per gene than SCR genes. This finding was attributed partly to the larger average size of the CSCWR genes and their ancient origin that allowed more point mutations to accumulate over a longer period of time. Rice and wheat are estimated to have evolved from a common ancestor approximately 50 million years ago^34^. Therefore these genes are an important resource for deciphering evolutionary history of rice. In addition, comparative analysis of SCR versus MCR gene based SNPs showed that MCR gene based SNPs have significantly lower average SNP call rates than SCR gene based SNPs.

An Affymetrix 44 K rice SNP array comprising 44,100 SNPs across the 12 rice chromosomes, having both genic and non-genic regions has been previously reported^10^. The average call rate in this chip was >92%, with up to 4.5% of missing data and >99% average pairwise concordance of technical replicates. Similarly, a 50 K (RiceSNP50)^13^ array, developed using Illumina Infinium platform, has 51,478 genome-wide SNPs, but only 68% of these SNPs were from genic regions. In contrast, our 50 K rice SNP chip (OsSNPnks) is an entirely genic chip, based specifically on SC genes. The average call rate is high (99.9%), with extremely little (<0.10) missing data and high assay reproducibility of 99.9%. According to our literature search, none of the existing rice SNP arrays or SNP arrays designed for any other plant or animal species are based on SC genes. Therefore, the concept of SC gene based chip may be used for other species, and this chip will have significant applications in diversity and evolutionary studies. The SNP chip (OsSNPnks) described here opens up a new avenue for rice researchers in different areas such as germplasm characterisation, phylogenetic analysis, background selection, association studies and QTL mapping for important agronomic and quality traits. SNP information on conserved SC genes will be useful for evolutionary and domestication related studies in cultivated rice and its wild relatives.

Furthermore, we incorporated SNPs from 194 AGCR genes in the chip; therefore geneticists have the opportunity to identify novel haplotypes of these genes useful for improving agronomic performance and nutritional quality. The utility of AGCR gene based SNPs in the 50 K rice chip was evaluated for background analysis in four mega rice varieties and their improved versions in terms of haplotype recovery of agronomically important genes. The results revealed highest recovery in Swarna-Sub1, which is quite popular in the flood-prone areas of Eastern India. In contrast, recovery in Samba Mahsuri-Sub1, which has not gained such wide acceptance, was only 52.6%, suggesting that more backcrossing is required to recover all the agronomic and quality traits of Samba Mahsuri-Sub1 for which it is popular amongst farmers. Background genome recovery analysis showed several interspersed segments of the donor parent genome in the backcross-derived submergence-tolerant rice cultivars, suggesting that the level of recombination resulting from double-crossover events in rice is reasonably high.

Application of this chip revealed exciting results, with several wild rice accessions showing proximity with the *Aus* group of cultivated rice, which may have been domesticated from this wild-rice group (Fig. 2). The *Aus* group of rice varieties is particularly adapted to upland drought conditions. Our genotyping chip will play a major role in understanding the evolutionary history of wild rice and domestication of cultivated rice. Varied applications of the 50 K (OsSNPnks) chip demonstrates that the chip is efficient and reliable tool for rice germplasm characterisation, association mapping, background selection and evolutionary studies.

## Methods

### Plant materials

A set of 192 diverse rice genotypes was used. The set included a collection of 86 wild rice accessions from the Gangetic Plain region of India (56 from Uttar Pradesh, 17 from Bihar, 1 from Gujarat, 5 from Himachal Pradesh, and 7 from the gene bank of National Bureau of Plant Genetic Resource, New Delhi), 93 cultivated rice varieties, and 13 landraces. Details of the wild rice accessions and rice cultivars are specified in Supplementary Table 3 and our wild rice database (http://nksingh.nationalprof.in/). To assess the performance of the 50 K (OsSNPnks) chip, the above mentioned rice germplasm was used for SNP genotyping, phylogenetic studies, and recipient genome background recovery analysis.

### Gene selection for the SNP array design

Total 18,980 rice genes, including 3,710 CSCWR, 14,959 SCR, 194 AGCR and 117 MCR genes, from the 12 rice chromosomes were identified and used for the SNP array design. To mine SC genes, all rice gene sequences were downloaded from the Rice Genome Annotation Project (http://rice.plantbiology.msu.edu/) database of pseudomolecule version 5.0. Pre-optimized BLASTN parameter was used for similarity search among the rice gene sequences^35^,^36^. Matched rice gene sequences were tabulated using BLAST Parser software^37^ and filtered in Microsoft Excel. Exactly matched gene sequences, identified at a bit-score threshold of 200, were extracted and compared with previously reported conserved SC gene common in wheat and rice^20^. After comparison, uniquely matched rice genes were retrieved from the combined dataset. AGCR genes were accessed from the in-house developed Cloned Gene Information System (http://125.18.242.19/plantgenomedb/cloned%20Gene/Index.html) database and through literature survey (Supplementary Table 5). The genome-wide MCR genes were acquired from the Rice Genome Annotation Project (http://rice.plantbiology.msu.edu/) to serve as controls for array designing. The MCR genes were identified using the BLASTN programme and were searched against a rice gene sequence database by using pre-optimised search parameters^35^. We retrieved total 1,881 copies of genes for 117 MCR genes, with an average of 16 copies per MCR gene (Supplementary Table 7).

### SNP identification and array design

Gene-based SNPs were identified *in silico* from different public databases *Oryza*SNP (http://oryzasnp.plantbiology.msu.edu/), Gramene (www.gramene.org) and by in-house multiple sequence alignment by using Bioedit^38^ and ClustalW software^39^, after considering 35 bases from either side of a SNP position. Initially, we designed 55,100 SNP assays, and *in silico* validation was performed using the AxiomGTv1 algorithm of APT, which generated an output score file containing p-convert values, signifying the SNP array quality and list of recommended and non-recommended SNP probes. For a high-quality SNP array design, we considered the SNPs with cut-off p-convert values of >0.30 and omitted those with p-convert values of <0.30. Based on the fore mentioned criteria, we fabricated rice Affymetrix chip with 50,051 high-quality SNPs.

### Target probe preparation and 50 K rice SNP array hybridisation

Rice genomic DNA was extracted from young green leaf tissue using the CTAB method^40^. DNA was quantified using a nano-drop spectrophotometer and read at 260/280 nm, and the quality of genomic DNA was checked on 1% agarose gel. For target probe preparation, 20 μL of gDNA was used for each DNA sample, and the concentration was 10 ng/μL (for a total 200 ng, gDNA in 20 μL) based on Affymetrix Axiom^®^ 2.0 Assay Manual. DNA amplification, fragmentation, chip hybridisation, single-base extension through DNA ligation and signal amplification were performed using the Affymetrix Axiom^®^ 2.0 Assay Manual Target Prep Protocol QRC (P/N 702990). Staining and scanning were performed on the GeneTitan^®^ Multi-Channel Instrument according to the manufacturer’s procedure (http://media.affymetrix.com/support/downloads/manuals/axiom_2_assay_auto_workflow_user_guide.pdf).

### SNP allele calling and data analysis

SNP genotypes were called using the Affymetrix Genotyping Console™v4.1 software package, which was designed to create genotype calls of CEL files. GTC requires information stored in library files to analyse the CEL files generated by GCOS Affymetrix software. SNPs with low call rates across all samples were removed from the dataset, and high-performing SNPs with a DQC of >0.85 and call rates of >95.0% were used for analyses. To evaluate the data quality the dataset was imported in other compatible formats, such as PLINK^41^ and text, was examined using two different software, APT with R package and SNPolisher (http://www.affymetrix.com).

### Haplotype-based phylogenetic tree construction

Total 128 diverse rice genotypes, including 83 wild rice accessions, 13 landraces, and 32 cultivated rice varieties, were used for phylogenetic analysis. SNP haplotypes were generated using TASSEL 3.2.1^42^ in a sliding window of a 5-nucleotide length. Based on the SNP haplotype analysis, a phylogenetic tree was constructed using an improved version of the neighbour-joining algorithm, and the tree was visualised using FigTree v1.4.0^43^.

## Additional Information

**How to cite this article**: Singh, N. *et al.* Single-copy gene based 50K SNP chip for genetic studies and molecular breeding in rice. *Sci. Rep.* **5**, 11600; doi: 10.1038/srep11600 (2015).

## Supplementary Material

Supplementary Information

Supplementary Table 1

Supplementary Table 2

Supplementary Table 3

Supplementary Table 4

Supplementary Table 5

Supplementary Table 6

Supplementary Table 7

## Figures and Tables

**Table 1:** Chromosome wise distribution of number of SNPs in different categories of rice genes in the Affymetrix 50 K rice SNP chip ‘OsSNPnks’.

**Chr.No.**	**CSCWR**		**SCR**		**AGCR**		**MCR**		**Total**
	**No. of Genes**	**No. of SNPs**	**No. of Genes**	**No. of SNPs**	**No. of Genes**	**No. of SNPs**	**No. of Genes**	**No. of SNPs**	
1	607	4664	2045	5197	29	154	1	1	10016
2	480	2718	1787	3250	13	59	7	40	6067
3	547	3000	1888	3852	27	172	4	20	7044
4	336	1548	1383	2054	18	170	3	4	3776
5	297	1914	1329	2236	16	109	11	38	4297
6	313	2292	1302	3050	22	145	4	15	5502
7	290	1098	1259	1147	9	48	12	41	2334
8	214	1022	1199	1454	16	102	0	0	2578
9	205	988	947	1337	7	41	13	97	2463
10	137	797	327	339	11	35	20	113	1284
11	143	999	789	1310	18	120	42	249	2678
12	141	1176	704	775	8	61	0	0	2012
Total	3710	22216	14959	26001	194	1216	117	618	50051

CSCWR, Conserved single-copy genes common to wheat and rice; SCR, Single-copy genes unique to rice; AGCR, Agronomically important cloned rice genes; MCR, Multi-copy rice genes.
