# Supplementary material for: Single-copy gene based 50 K SNP chip for genetic studies and molecular breeding in rice
Source: Sci Rep. 2015 Jun 26;5:11600. doi: 10.1038/srep11600 (PMC4481378; doi:10.1038/srep11600)
Supplement: Supplementary Information [file srep11600-s1.doc]

**Single-copy gene based 50K SNP chip for genetic studies and molecular breeding in rice**

Nisha Singh^1,2^, Pawan Kumar Jayaswal^1^, Kabita Panda^1^, Paritra Mandal^1^, Vinod Kumar^1^, Balwant Singh^1^, Shefali Mishra^1^, Yashi Singh^1^, Renu Singh^1^, Vandna Rai^1^, Anita Gupta^2^, Tilak Raj Sharma^1^ & Nagendra Kumar Singh^1^

^1^National Research Centre on Plant Biotechnology, Indian Agricultural Research Institute, New Delhi 110012, India

^2^Rayat and Bahra Institute of Engineering and Bio-Technology, Mohali, Punjab Technical University, Jalandhar 140104, India

Correspondence should be addressed to N.K.S. ([nksingh4@gmail.com](mailto:nksingh4@gmail.com), [nksingh@nrcpb.org](mailto:nksingh@nrcpb.org)); Mob. +91-9911268915; Tel. +91-11-2584-1186; Fax +91-11-2584-3984

**Supplementary Figure**


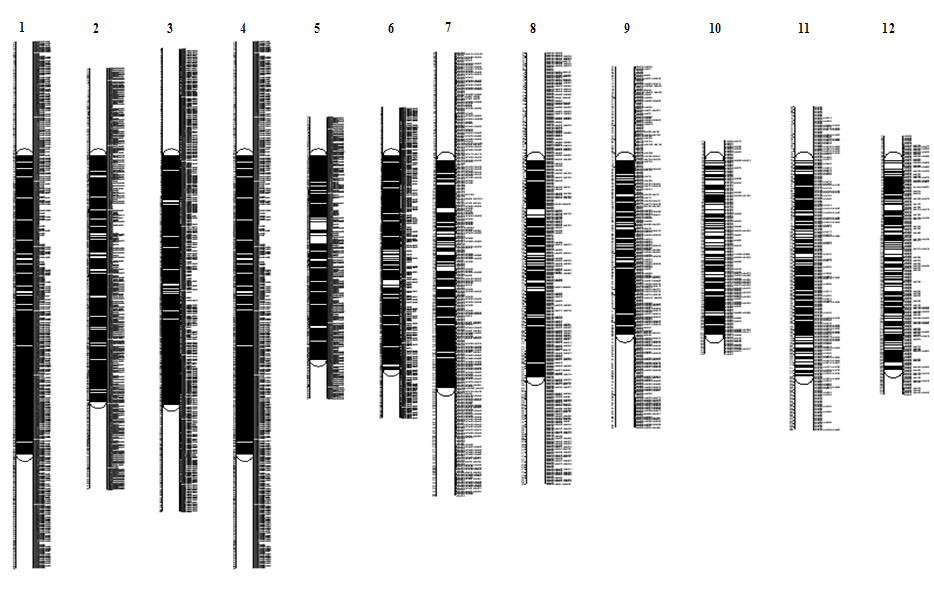


**Supplementary Figure 1:** Physical map of the 12 rice chromosomes showing high density genome-wide coverage of the single-copy rice genes used in the ‘OsSNPnks’ chip design.

**Supplementary Table Legends**

**Supplementary table 1|** 50K SNP assay information including Affymetrix Probe Id and NRCPB SNP Id containing gene number, chromosome pseudomolecule base positions (last five digits) and SNP flanking DNA sequence.

**Supplementary table 2|** 50K SNP genotyping data quality of 192 rice samples with respect to call rate, heterozygosity and DQC.

**Supplementary Table 3|** List of 192 rice genotypes used for the validation and application of 50K rice SNP chip, including 83 accessions of wild rice collected mostly from the Indo-Gangatic region (prefixed NKSWR) and 7 accessions of *O. nivara* and *O. rufipogon* obtained from NBPGR gene bank. Genotypes are arranged and color coded to match the haplotype based phylogenetic grouping in Figure 2; Group I (blue) includes wild rice with exception of ‘*Sathi’*, group II (green) includes wild rice along with *Aus* type cultivated rice, Group III (red) includes wild rice along with *Indica* type rice cultivars and group IV (cyan) includes two *Japonica* rice cultivars. Genotypes excluded from the phylogenetic analysis are shown in black font towards the end of the Table.

**Supplementary Table 4|** Analysis of chromosome-wise percentage of background similarity with the recipient parent in submergence tolerant rice varieties based on the 50K chip data.

**Supplementary Table 5|** List of agronomically important cloned rice (AGCR) genes incorporated in "OsSNPnks" 50K SNP chip with trait information and annotated molecualr function.

**Supplementary Table 6|** List of AGCR (agronomically important cloned rice) genes with donor type alleles in Swarna-sub1 out of total 194 AGCR genes assayed in the 50K SNP chip.

**Supplementary Table 7|** List of MCR genes incorporated in 50K “OsSNPnks” chip corresponding with their copy numbers and putative gene functions.
