# Supplementary material for: Single-copy gene based 50 K SNP chip for genetic studies and molecular breeding in rice
Source: Sci Rep. 2015 Jun 26;5:11600. doi: 10.1038/srep11600 (PMC4481378; doi:10.1038/srep11600)
Supplement: Supplementary Table 2 [file srep11600-s3.doc]

**Supplementary table 2|** 50K SNP genotyping data quality of 192 rice samples with respect to call rate, heterozygosity and DQC

| **S. No.** | **Genotypes** | **Call rate** | **Hetero_rate** | **Hom_rate** | **DQC Value** |
| --- | --- | --- | --- | --- | --- |
| 1 | NKSWR1 | 99.5 | 1.4 | 98.1 | 0.92 |
| 2 | NKSWR10 | 99.6 | 1.2 | 98.4 | 0.93 |
| 3 | NKSWR100 | 99.6 | 1.4 | 98.2 | 0.95 |
| 4 | NKSWR101 | 99.5 | 1.6 | 98.0 | 0.95 |
| 5 | NKSWR106 | 95.0 | 10.1 | 84.9 | 0.97 |
| 6 | NKSWR11 | 98.9 | 1.2 | 97.7 | 0.93 |
| 7 | NKSWR110 | 99.6 | 1.5 | 98.1 | 0.95 |
| 8 | NKSWR111 | 99.6 | 1.1 | 98.5 | 0.98 |
| 9 | NKSWR112 | 99.4 | 1.4 | 98.0 | 0.95 |
| 10 | NKSWR118 | 99.5 | 1.4 | 98.1 | 0.95 |
| 11 | NKSWR12 | 98.3 | 1.7 | 96.7 | 0.97 |
| 12 | NKSWR121 | 99.4 | 1.6 | 97.8 | 0.97 |
| 13 | NKSWR122 | 99.3 | 1.3 | 98.0 | 0.93 |
| 14 | NKSWR123 | 99.7 | 1.0 | 98.7 | 0.97 |
| 15 | NKSWR124 | 99.1 | 18.2 | 81.0 | 0.97 |
| 16 | NKSWR125 | 99.3 | 2.2 | 97.1 | 0.94 |
| 17 | NKSWR127 | 99.1 | 1.7 | 97.5 | 0.94 |
| 18 | NKSWR13 | 99.5 | 1.2 | 98.2 | 0.93 |
| 19 | NKSWR133 | 95.2 | 13.4 | 81.8 | 0.98 |
| 20 | NKSWR139 | 98.6 | 24.9 | 73.6 | 0.98 |
| 21 | NKSWR14 | 95.5 | 27.8 | 67.7 | 0.98 |
| 22 | NKSWR145 | 99.6 | 1.5 | 98.1 | 0.97 |
| 23 | NKSWR15 | 98.2 | 2.0 | 96.2 | 0.97 |
| 24 | NKSWR155 | 99.3 | 10.1 | 89.1 | 0.97 |
| 25 | NKSWR156 | 99.5 | 1.7 | 97.8 | 0.96 |
| 26 | NKSWR16 | 99.4 | 1.1 | 98.3 | 0.93 |
| 27 | NKSWR168 | 99.6 | 1.2 | 98.4 | 0.98 |
| 28 | NKSWR169 | 99.6 | 0.9 | 98.7 | 0.97 |
| 29 | NKSWR17 | 99.5 | 1.2 | 98.3 | 0.93 |
| 30 | NKSWR170 | 99.6 | 0.9 | 98.7 | 0.97 |
| 31 | NKSWR171 | 99.7 | 1.1 | 98.6 | 0.98 |
| 32 | NKSWR173 | 98.8 | 38.2 | 60.6 | 0.98 |
| 33 | NKSWR18 | 99.4 | 1.2 | 98.3 | 0.94 |
| 34 | NKSWR188 | 98.3 | 10.9 | 87.4 | 0.96 |
| 35 | NKSWR19 | 99.6 | 1.5 | 98.1 | 0.94 |
| 36 | NKSWR 191 | 93.6 | 13.7 | 79.9 | 0.93 |
| 37 | NKSWR199 | 99.3 | 9.6 | 89.7 | 0.98 |
| 38 | NKSWR20 | 99.7 | 0.9 | 98.8 | 0.98 |
| 39 | NKSWR21 | 99.6 | 1.6 | 98.0 | 0.94 |
| 40 | NKSWR213 | 99.6 | 1.5 | 98.1 | 0.96 |
| 41 | NKSWR22 | 99.4 | 1.2 | 98.2 | 0.93 |
| 42 | NKSWR23 | 99.6 | 1.3 | 98.3 | 0.96 |
| 43 | NKSWR234 | 99.3 | 1.5 | 97.8 | 0.98 |
| 44 | NKSWR25 | 99.6 | 1.5 | 98.0 | 0.95 |
| 45 | NKSWR26 | 99.6 | 1.1 | 98.5 | 0.93 |
| 46 | NKSWR27 | 99.4 | 1.4 | 98.0 | 0.92 |
| 47 | NKSWR28 | 99.4 | 1.6 | 97.7 | 0.95 |
| 48 | NKSWR29 | 99.6 | 1.2 | 98.3 | 0.94 |
| 49 | NKSWR30 | 99.0 | 1.8 | 97.2 | 0.94 |
| 50 | NKSWR31 | 99.6 | 0.9 | 98.7 | 0.97 |
| 51 | NKSWR32 | 99.7 | 1.0 | 98.7 | 0.95 |
| 52 | NKSWR33 | 99.5 | 1.2 | 98.2 | 0.92 |
| 53 | NKSWR34 | 99.6 | 1.3 | 98.3 | 0.95 |
| 54 | NKSWR35 | 99.5 | 1.2 | 98.3 | 0.94 |
| 55 | NKSWR37 | 99.5 | 0.9 | 98.7 | 0.97 |
| 56 | NKSWR4 | 99.2 | 1.4 | 97.8 | 0.93 |
| 57 | NKSWR48 | 99.2 | 4.1 | 95.1 | 0.98 |
| 58 | NKSWR5 | 99.5 | 1.2 | 98.3 | 0.94 |
| 59 | NKSWR53 | 99.4 | 1.3 | 98.1 | 0.95 |
| 60 | NKSWR56 | 99.3 | 1.6 | 97.8 | 0.95 |
| 61 | NKSWR6 | 99.3 | 1.5 | 97.8 | 0.93 |
| 62 | NKSWR62 | 99.3 | 1.4 | 97.9 | 0.93 |
| 63 | NKSWR63 | 99.6 | 1.3 | 98.1 | 0.97 |
| 64 | NKSWR63 | 99.6 | 1.3 | 98.1 | 0.97 |
| 65 | NKSWR63 | 99.6 | 1.3 | 98.1 | 0.97 |
| 66 | NKSWR64 | 98.5 | 7.1 | 91.4 | 0.97 |
| 67 | NKSWR67 | 99.6 | 1.1 | 98.4 | 0.97 |
| 68 | NKSWR7 | 99.7 | 1.3 | 98.4 | 0.97 |
| 69 | NKSWR72 | 99.3 | 3.8 | 95.5 | 0.98 |
| 70 | NKSWR73 | 99.6 | 1.5 | 98.1 | 0.96 |
| 71 | NKSWR74 | 98.6 | 14.3 | 84.3 | 0.97 |
| 72 | NKSWR75 | 99.6 | 0.8 | 98.8 | 0.98 |
| 73 | NKSWR79 | 99.3 | 1.4 | 97.8 | 0.93 |
| 74 | NKSWR8 | 99.2 | 1.6 | 97.7 | 0.94 |
| 75 | NKSWR84 | 99.5 | 2.4 | 97.1 | 0.98 |
| 76 | NKSWR9 | 99.6 | 1.5 | 98.1 | 0.93 |
| 77 | NKSWR91 | 99.5 | 1.0 | 98.5 | 0.98 |
| 78 | NKSWR93 | 99.5 | 1.5 | 98.0 | 0.94 |
| 79 | *O.nivara* 330630 | 99.6 | 1.0 | 98.6 | 0.96 |
| 80 | *O.nivara* 330639 | 99.6 | 1.0 | 98.6 | 0.95 |
| 81 | *O.nivara* 330641 | 98.2 | 1.6 | 96.6 | 0.96 |
| 82 | *O.nivara* 330642 | 99.7 | 1.0 | 98.7 | 0.97 |
| 83 | *O.rufipogon* 336687 | 98.8 | 2.0 | 96.3 | 0.99 |
| 84 | *O.rufipogon* 336687 | 98.8 | 2.0 | 96.3 | 0.99 |
| 85 | *O.rufipogon* 336708 | 99.2 | 1.0 | 98.2 | 0.97 |
| 86 | *O.nivara* 330641 | 98.2 | 1.6 | 96.6 | 0.96 |
| 87 | 13-Amitha | 97.0 | 7.7 | 89.3 | 0.99 |
| 88 | 95-Amitha | 99.8 | 2.6 | 97.2 | 0.96 |
| 89 | 159-B | 98.8 | 1.3 | 97.5 | 0.98 |
| 90 | 862-B | 99.6 | 0.9 | 98.7 | 0.98 |
| 91 | ADT39 | 99.8 | 0.7 | 99.1 | 0.98 |
| 92 | ADT45 | 99.6 | 1.0 | 98.7 | 0.98 |
| 93 | ADT46 | 99.6 | 0.9 | 98.7 | 0.99 |
| 94 | APO | 99.5 | 1.0 | 98.5 | 0.97 |
| 95 | B-6 | 99.6 | 0.9 | 98.8 | 0.99 |
| 96 | CR1009 Sub1 | 99.5 | 1.0 | 98.5 | 0.98 |
| 97 | CSR11 | 99.6 | 5.4 | 94.2 | 0.98 |
| 98 | CSR1_Bulk S | 97.2 | 15.2 | 82.0 | 0.99 |
| 99 | CSR11_Bulk T | 98.0 | 15.7 | 82.3 | 0.98 |
| 100 | CSR27 | 99.5 | 1.0 | 98.6 | 0.98 |
| 101 | CSR2_Bulk S | 97.0 | 18.2 | 78.8 | 0.99 |
| 102 | CSR27_Bulk T | 98.2 | 15.4 | 82.8 | 0.99 |
| 103 | Dihawan-1_NKSLR11 | 99.7 | 0.9 | 98.8 | 0.99 |
| 104 | Dihawan_NKSLR11 | 99.7 | 0.9 | 98.8 | 0.99 |
| 105 | Amitha_bulk | 96.2 | 27.5 | 68.7 | 0.99 |
| 106 | Dudhaladu_NKSLR17 | 95.9 | 2.0 | 93.9 | 0.97 |
| 107 | FL478 | 99.7 | 1.0 | 98.8 | 0.97 |
| 108 | FR13A | 99.6 | 1.0 | 98.6 | 0.97 |
| 109 | 1121+Pi54+Piz5-1 | 99.2 | 3.2 | 96.0 | 0.98 |
| 110 | 1121+Pi54+Piz5-2 | 99.4 | 3.3 | 96.1 | 0.95 |
| 111 | 1121+Saltol-2 | 99.5 | 1.8 | 97.7 | 0.97 |
| 112 | 1121+xa13+Xa21-1 | 98.5 | 11.3 | 87.2 | 0.97 |
| 113 | 1121+xa13+Xa21-2 | 98.1 | 27.7 | 70.4 | 0.98 |
| 114 | 1121+Xa38-1 | 99.6 | 6.2 | 93.4 | 0.97 |
| 115 | 1121+Xa38-2 | 99.7 | 2.4 | 97.3 | 0.97 |
| 116 | 1401+Pi54+Piz5-1 | 99.6 | 4.5 | 95.1 | 0.97 |
| 117 | 1401+Pi54+Piz5-2 | 99.6 | 2.8 | 96.8 | 0.98 |
| 118 | 1401+xa13+Xa21-1 | 99.5 | 1.1 | 98.3 | 0.98 |
| 119 | 1401+xa13+Xa21-2 | 99.5 | 1.3 | 98.3 | 0.97 |
| 120 | Gayatri | 99.6 | 0.8 | 98.9 | 0.98 |
| 121 | BML-35-1 | 99.5 | 0.9 | 98.6 | 0.98 |
| 122 | BML-38 | 99.4 | 1.3 | 98.1 | 0.98 |
| 123 | Gothwa_NKSLR1 | 99.6 | 1.1 | 98.5 | 0.98 |
| 124 | P-1460 | 99.7 | 3.9 | 95.8 | 0.97 |
| 125 | P-1602 | 99.5 | 2.6 | 96.9 | 0.97 |
| 126 | P-1609 | 98.8 | 1.8 | 97.0 | 0.96 |
| 127 | PB1+Pi5-1 | 95.8 | 2.8 | 93.0 | 0.99 |
| 128 | PB1+Pi5-2 | 99.6 | 1.9 | 97.7 | 0.99 |
| 129 | PB1+Pi5+Pi9-1 | 99.8 | 1.2 | 98.6 | 0.97 |
| 130 | PB1+Pi5+Pi9-2 | 99.7 | 1.4 | 98.4 | 0.96 |
| 131 | PB1+Pi9-1 | 96.7 | 20.6 | 76.1 | 0.99 |
| 132 | PB1+Pi9-2 | 99.5 | 1.0 | 98.5 | 0.96 |
| 133 | PB-1401 | 97.5 | 6.7 | 90.8 | 0.97 |
| 134 | PR114 | 99.4 | 1.2 | 98.2 | 0.97 |
| 135 | Taipie 309 | 99.4 | 1.0 | 98.4 | 0.96 |
| 136 | HG_Bulk | 95.2 | 23.3 | 71.9 | 0.99 |
| 137 | HUR105 | 99.5 | 0.8 | 98.7 | 0.98 |
| 138 | IR64-Sub1 | 99.7 | 1.5 | 98.1 | 0.98 |
| 139 | IR64 | 99.5 | 1.6 | 97.9 | 0.97 |
| 140 | IR81896-B-B-148 | 99.6 | 0.9 | 98.7 | 0.98 |
| 141 | IR81896-B-B-195 | 99.7 | 0.8 | 98.9 | 0.98 |
| 142 | IR86918-B-305 | 99.6 | 1.4 | 98.2 | 0.98 |
| 143 | IR87728-59-B-B | 99.5 | 0.9 | 98.6 | 0.98 |
| 144 | IR87728-75-B-B | 99.7 | 0.9 | 98.8 | 0.99 |
| 145 | IR87728-367-B-B | 99.7 | 0.9 | 98.8 | 0.98 |
| 146 | JADHAN_NKSLR15 | 99.7 | 0.8 | 98.9 | 0.98 |
| 147 | Kajarahwa_NKSLR2 | 99.6 | 1.1 | 98.5 | 0.99 |
| 148 | Karahani_NKSLR7 | 98.6 | 1.9 | 96.7 | 0.98 |
| 149 | Kariyawa_NKSLR10 | 96.3 | 11.7 | 84.6 | 0.98 |
| 150 | LG_Bulk | 97.3 | 19.8 | 77.5 | 0.99 |
| 151 | MI48 | 99.6 | 0.9 | 98.8 | 0.99 |
| 152 | MTU1010 | 99.6 | 0.8 | 98.8 | 0.99 |
| 153 | MTU1075 | 99.6 | 0.9 | 98.7 | 0.98 |
| 154 | Nagina22 | 99.5 | 1.0 | 98.5 | 0.97 |
| 155 | NPT11 | 96.5 | 21.9 | 74.6 | 0.98 |
| 156 | PB1 | 99.7 | 0.9 | 98.8 | 0.96 |
| 157 | PB1+Pi5-3 | 98.7 | 2.1 | 96.6 | 0.97 |
| 158 | Pokkali | 99.7 | 0.9 | 98.9 | 0.97 |
| 159 | Pooja | 99.6 | 0.8 | 98.9 | 0.98 |
| 160 | PR114 | 99.7 | 0.8 | 98.9 | 0.99 |
| 161 | Pratikshya | 99.6 | 0.9 | 98.8 | 0.99 |
| 162 | Pusa44 | 99.8 | 0.8 | 99.0 | 0.98 |
| 163 | R1 | 99.7 | 2.4 | 97.3 | 0.98 |
| 164 | R2 | 99.5 | 3.5 | 96.0 | 0.96 |
| 165 | R3 | 99.7 | 1.0 | 98.7 | 0.97 |
| 166 | R4 | 99.6 | 1.0 | 98.6 | 0.97 |
| 167 | R5 | 99.6 | 2.2 | 97.3 | 0.98 |
| 168 | R7 | 99.7 | 1.3 | 98.5 | 0.98 |
| 169 | R10 | 99.6 | 3.9 | 95.6 | 0.98 |
| 170 | R11 | 99.1 | 6.9 | 92.2 | 0.98 |
| 171 | R13 | 99.1 | 3.5 | 95.6 | 0.98 |
| 172 | R14 | 99.6 | 1.4 | 98.2 | 0.98 |
| 173 | R15 | 99.6 | 1.5 | 98.2 | 0.96 |
| 174 | R16 | 99.7 | 0.9 | 98.7 | 0.97 |
| 175 | R17 | 99.7 | 1.3 | 98.4 | 0.98 |
| 176 | R18 | 99.7 | 1.6 | 98.1 | 0.97 |
| 177 | R19 | 99.7 | 2.1 | 97.6 | 0.97 |
| 178 | Rajendra Mahsuri | 99.7 | 0.9 | 98.7 | 0.98 |
| 179 | Rajendra Mahsuri_1 | 99.7 | 0.9 | 98.7 | 0.98 |
| 180 | Ranjit | 99.7 | 0.8 | 98.8 | 0.98 |
| 181 | RPBIO | 99.7 | 0.9 | 98.9 | 0.97 |
| 182 | Sambha Mahsuri Sub1 | 99.7 | 0.8 | 98.8 | 0.98 |
| 183 | Sambha Mahsuri | 99.3 | 1.6 | 97.7 | 0.98 |
| 184 | Sarjoo52 | 99.7 | 0.9 | 98.8 | 0.98 |
| 185 | Sathi_NKSLR9 | 99.4 | 1.4 | 98.1 | 0.95 |
| 186 | Sathwa_NKSLR3 | 99.3 | 1.2 | 98.2 | 0.97 |
| 187 | CR1009 | 99.6 | 1.5 | 98.2 | 0.98 |
| 188 | Singhara_NKSLR5 | 99.6 | 1.0 | 98.6 | 0.99 |
| 189 | Sugapankh_NKSLR14 | 98.2 | 17.3 | 80.9 | 0.99 |
| 190 | Swarna | 99.7 | 0.8 | 98.9 | 0.97 |
| 191 | Swarna Sub1 | 99.7 | 0.8 | 98.9 | 0.98 |
| 192 | Tundhiya_NKSLR6 | 99.4 | 1.1 | 98.3 | 0.98 |

All rice genotypes showing call rates >95.0%, except one which showed a call rate of 93.6
