# Supplementary material for: Single-copy gene based 50 K SNP chip for genetic studies and molecular breeding in rice
Source: Sci Rep. 2015 Jun 26;5:11600. doi: 10.1038/srep11600 (PMC4481378; doi:10.1038/srep11600)
Supplement: Supplementary Table 3 [file srep11600-s4.doc]

**Supplementary Table 3|** List of 192 rice genotypes used for the validation and application of 50K rice SNP chip, including 83 accessions of wild rice collected mostly from the Indo-Gangatic region (prefixed NKSWR) and 7 accessions of *O. nivara* and *O. rufipogon* obtained from NBPGR gene bank. Genotypes are arranged and color coded to match the haplotype based phylogenetic grouping in Figure 2; Group I (blue) includes wild rice with exception of ‘*Sathi’*, group II (green) includes wild rice along with *Aus* type cultivated rice, Group III (red) includes wild rice along with *Indica* type rice cultivars and group IV (cyan) includes two *Japonica* rice cultivars. Genotypes excluded from the phylogenetic analysis are shown in black font towards the end of the Table.

| S. No | Genotypes | Source | Collection site (for wild rice) |
| --- | --- | --- | --- |
| 1 | NKSWR1 | New collection | Varanasi, Uttar Pradesh |
| 2 | NKSWR4 | New collection | Mirzapur, Uttar Pradesh |
| 3 | NKSWR5 | New collection | Mirzapur, Uttar Pradesh |
| 4 | NKSWR6 | New collection | Mirzapur, Uttar Pradesh |
| 5 | NKSWR8 | New collection | Mirzapur, Uttar Pradesh |
| 6 | NKSWR9 | New collection | Mirzapur, Uttar Pradesh |
| 7 | NKSWR11 | New collection | Mirzapur, Uttar Pradesh |
| 8 | NKSWR13 | New collection | Mirzapur, Uttar Pradesh |
| 9 | NKSWR14 | New collection | Mirzapur, Uttar Pradesh |
| 10 | NKSWR15 | New collection | Mirzapur, Uttar Pradesh |
| 11 | NKSWR16 | New collection | Mirzapur, Uttar Pradesh |
| 12 | NKSWR25 | New collection | Chandauli, Uttar Pradesh |
| 13 | NKSWR26 | New collection | Chandauli, Uttar Pradesh |
| 14 | NKSWR27 | New collection | Chandauli, Uttar Pradesh |
| 15 | NKSWR29 | New collection | Chandauli, Uttar Pradesh |
| 16 | NKSWR30 | New collection | Chandauli, Uttar Pradesh |
| 17 | NKSWR31 | New collection | Chandauli, Uttar Pradesh |
| 18 | NKSWR33 | New collection | Chandauli, Uttar Pradesh |
| 19 | NKSWR34 | New collection | Chandauli, Uttar Pradesh |
| 20 | NKSWR35 | New collection | Ghazipur, Uttar Pradesh |
| 21 | NKSWR36 | New collection | Ghazipur, Uttar Pradesh |
| 22 | NKSWR100 | New collection | Ghazipur, Uttar Pradesh |
| 23 | NKSWR18 | New collection | Sonbhadra, Uttar Pradesh |
| 24 | NKSWR19 | New collection | Sonbhadra, Uttar Pradesh |
| 25 | NKSWR21 | New collection | Sonbhadra, Uttar Pradesh |
| 26 | NKSWR22 | New collection | Sonbhadra, Uttar Pradesh |
| 27 | NKSWR23 | New collection | Sonbhadra, Uttar Pradesh |
| 28 | NKSWR56 | New collection | Ballia, Uttar Pradesh |
| 29 | NKSWR62 | New collection | Ballia, Uttar Pradesh |
| 30 | NKSWR63 | New collection | Ballia, Uttar Pradesh |
| 31 | NKSWR101 | New collection | Ballia, Uttar Pradesh |
| 32 | NKSWR64 | New collection | Mau, Uttar Pradesh |
| 33 | NKSWR67 | New collection | Mau, Uttar Pradesh |
| 34 | NKSWR73 | New collection | Azamgarh, Uttar Pradesh |
| 35 | NKSWR74 | New collection | Azamgarh, Uttar Pradesh |
| 36 | NKSWR75 | New collection | Azamgarh, Uttar Pradesh |
| 37 | NKSWR84 | New collection | Azamgarh, Uttar Pradesh |
| 38 | NKSWR188 | New collection | Sitapur, Uttar Pradesh |
| 39 | NKSWR191 | New collection | Hardoi, Uttar Pradesh |
| 40 | NKSWR199 | New collection | Unnao, Uttar Pradesh |
| 41 | NKSWR213 | New collection | Kushi Nagar, Uttar Pradesh |
| 42 | NKSWR139 | New collection | Aurangabad, Bihar |
| 43 | NKSWR106 | New collection | Kaimur, Bihar |
| 44 | NKSWR110 | New collection | Kaimur, Bihar |
| 45 | NKSWR111 | New collection | Kaimur, Bihar |
| 46 | NKSWR112 | New collection | Kaimur, Bihar |
| 47 | NKSWR122 | New collection | Bhojpur, Bihar |
| 48 | NKSWR123 | New collection | Bhojpur, Bihar |
| 49 | NKSWR124 | New collection | Bhojpur, Bihar |
| 50 | NKSWR155 | New collection | Bhojpur, Bihar |
| 51 | NKSWR156 | New collection | Bhojpur, Bihar |
| 52 | NKSWR118 | New collection | Rohtas, Bihar |
| 53 | NKSWR127 | New collection | Rohtas, Bihar |
| 54 | NKSWR121 | New collection | Buxar, Bihar |
| 55 | NKSWR145 | New collection | Gaya, Bihar |
| 56 | Sathi_NKSLR9 | New collection | Mau, Uttar Pradesh |
| 57 | *O. nivara* 330641 | NBPGR, New Delhi | 24 Parganas, West Bengal |
| 58 | *O. rufipogon* 336687 | NBPGR, New Delhi | Balangir, Odisha |
| 59 | NKSWR7 | New collection | Mirzapur, Uttar Pradesh |
| 60 | NKSWR10 | New collection | Mirzapur, Uttar Pradesh |
| 61 | NKSWR12 | New collection | Mirzapur, Uttar Pradesh |
| 62 | NKSWR28 | New collection | Chandauli, Uttar Pradesh |
| 63 | NKSWR32 | New collection | Chandauli, Uttar Pradesh |
| 64 | NKSWR91 | New collection | Ghazipur, Uttar Pradesh |
| 65 | NKSWR93 | New collection | Ghazipur, Uttar Pradesh |
| 66 | NKSWR37 | New collection | Ghazipur, Uttar Pradesh |
| 67 | NKSWR72 | New collection | Azamgarh, Uttar Pradesh |
| 68 | NKSWR17 | New collection | Sonbhadra, Uttar Pradesh |
| 69 | NKSWR168 | New collection | Mandi, Himachal Pradesh |
| 70 | NKSWR170 | New collection | Kangra, Himachal Pradesh |
| 71 | NKSWR171 | New collection | Kangra, Himachal Pradesh |
| 72 | NKSWR173 | New collection | Kangra, Himachal Pradesh |
| 73 | NKSWR125 | New collection | Bhojpur, Bihar |
| 74 | NKSWR133 | New collection | Aurangabad, Bihar |
| 75 | NKSWR234 | New collection | Navsari, Gujarat |
| 76 | *O. nivara* 330630 | NBPGR, New Delhi | 24 Parganas, West Bengal |
| 77 | *O. nivara* 330639 | NBPGR, New Delhi | 24 Parganas, West Bengal |
| 78 | *O. nivara* 330642 | NBPGR, New Delhi | 24 Parganas, West Bengal |
| 79 | *O. rufipogon* 336708 | NBPGR, New Delhi | Raigad, Maharashtra |
| 80 | Tundahiya_NKSLR6 | New collection | Ballia ,Uttar Pradesh |
| 81 | Dihawan-1_NKSLR11-1 | New collection | Ballia ,Uttar Pradesh |
| 82 | Kajarhwa_NKSLR2 | New collection | Ballia ,Uttar Pradesh |
| 83 | Karahani_NKSLR7 | New collection | Ballia ,Uttar Pradesh |
| 84 | FR13A | NRCPB, New Delhi |  |
| 85 | Nagina22 | NRCPB, New Delhi |  |
| 86 | NKSWR169 | New collection | Mandi, Himachal Pradesh |
| 87 | NKSWR20 | New collection | Sonbhadra, Uttar Pradesh |
| 88 | NKSWR48 | New collection | Ballia, Uttar Pradesh |
| 89 | NKSWR53 | New collection | Ballia, Uttar Pradesh |
| 90 | NKSWR79 | New collection | Azamgarh, Uttar Pradesh |
| 91 | Dihawan_NKSLR11 | New collection | Ballia, Uttar Pradesh |
| 92 | Dudhaladu_NKSLR17 | New collection | Ballia, Uttar Pradesh |
| 93 | Gothawa_NKSLR1 | New collection | Ballia, Uttar Pradesh |
| 94 | Jadahan_NKSLR15 | New collection | Mau, Uttar Pradesh |
| 95 | Kajarahwa_NKSLR2 | New collection | Ballia, Uttar Pradesh |
| 96 | Kariyawa_NKSLR10 | New collection | Ballia, Uttar Pradesh |
| 97 | Sathwa_NKSLR3 | New collection | Ballia, Uttar Pradesh |
| 98 | Singhara_NKSLR5 | New collection | Ballia, Uttar Pradesh |
| 99 | Sugapankh_NKSLR14 | New collection | Ballia, Uttar Pradesh |
| 100 | ADT39 | NRCPB, New Delhi |  |
| 101 | ADT45 | NRCPB, New Delhi |  |
| 102 | ADT46 | NRCPB, New Delhi |  |
| 103 | APO | NRCPB, New Delhi |  |
| 104 | CR1009 | NRCPB, New Delhi |  |
| 105 | CR1009 SUB1 | NRCPB, New Delhi |  |
| 106 | CSR11 | NRCPB, New Delhi |  |
| 107 | CSR27 | NRCPB, New Delhi |  |
| 108 | FL478 | NRCPB, New Delhi |  |
| 109 | HUR105 | NRCPB, New Delhi |  |
| 110 | IR64 | NRCPB, New Delhi |  |
| 111 | MI48 | NRCPB, New Delhi |  |
| 112 | NPT11 | NRCPB, New Delhi |  |
| 113 | PB1 | NRCPB, New Delhi |  |
| 114 | POKKALI | NRCPB, New Delhi |  |
| 115 | Pooja | NRCPB, New Delhi |  |
| 116 | PR114 | NRCPB, New Delhi |  |
| 117 | Pratikshya | NRCPB, New Delhi |  |
| 118 | Pusa44 | NRCPB, New Delhi |  |
| 119 | Rajendra Mahsuri | NRCPB, New Delhi |  |
| 120 | Ranjit | NRCPB, New Delhi |  |
| 121 | Sambha Mahsuri | NRCPB, New Delhi |  |
| 122 | Sambha Mahsuri Sub1 | NRCPB, New Delhi |  |
| 123 | Sarjoo52 | NRCPB, New Delhi |  |
| 124 | Swarna | NRCPB, New Delhi |  |
| 125 | SwarnaSub1 | NRCPB, New Delhi |  |
| 126 | Gayatri | IARI, New Delhi |  |
| 127 | Taipie309 | NRCPB, New Delhi |  |
| 128 | Nipponbare | NRCPB, New Delhi |  |
| 129 | RPBIO | NRCPB, New Delhi |  |
| 130 | CSR11 Bulk S | NRCPB, New Delhi |  |
| 131 | CSR11 Bulk T | NRCPB, New Delhi |  |
| 132 | CSR27 Bulk S | NRCPB, New Delhi |  |
| 133 | CSR27 Bulk T | NRCPB, New Delhi |  |
| 134 | B-6 | NRCPB, New Delhi |  |
| 135 | MTU1010 | NRCPB, New Delhi |  |
| 136 | MTU1075 | NRCPB, New Delhi |  |
| 137 | 159-B | NRCPB, New Delhi |  |
| 138 | 862-B | NRCPB, New Delhi |  |
| 139 | IR81896-B-B-148 | NRCPB, New Delhi |  |
| 140 | IR81896-B-B-195 | NRCPB, New Delhi |  |
| 141 | IR86918-B-305 | NRCPB, New Delhi |  |
| 142 | IR87728-367-B-B | NRCPB, New Delhi |  |
| 143 | IR87728-59-B-B | NRCPB, New Delhi |  |
| 144 | IR87728-75-B-B | NRCPB, New Delhi |  |
| 145 | LG Bulk | NRCPB, New Delhi |  |
| 146 | HG Bulk | NRCPB, New Delhi |  |
| 147 | 13-A | NRCPB, New Delhi |  |
| 148 | 95-A | NRCPB, New Delhi |  |
| 149 | R1 | IGKV, Raipur |  |
| 150 | R10 | IGKV, Raipur |  |
| 151 | R11 | IGKV, Raipur |  |
| 152 | R13 | IGKV, Raipur |  |
| 153 | R14 | IGKV, Raipur |  |
| 154 | R15 | IGKV, Raipur |  |
| 155 | R16 | IGKV, Raipur |  |
| 156 | R17 | IGKV, Raipur |  |
| 157 | R18 | IGKV, Raipur |  |
| 158 | R19 | IGKV, Raipur |  |
| 159 | R2 | IGKV, Raipur |  |
| 160 | R3 | IGKV, Raipur |  |
| 161 | R4 | IGKV, Raipur |  |
| 162 | R5 | IGKV, Raipur |  |
| 163 | R7 | IGKV, Raipur |  |
| 164 | P-1460 | IARI, New Delhi |  |
| 165 | P-1602 | IARI, New Delhi |  |
| 166 | P-1609 | IARI, New Delhi |  |
| 167 | PB1+Pi5+Pi9-1 | IARI, New Delhi |  |
| 168 | PB1+Pi5+Pi9-2 | IARI, New Delhi |  |
| 169 | PB1+Pi5-1 | IARI, New Delhi |  |
| 170 | PB1+Pi5-2 | IARI, New Delhi |  |
| 171 | PB1+Pi9-1 | IARI, New Delhi |  |
| 172 | PB1+Pi9-2 | IARI, New Delhi |  |
| 173 | PB-1401 | IARI, New Delhi |  |
| 174 | PR114 | IARI, New Delhi |  |
| 175 | 1121+Pi54+Piz5-1 | IARI, New Delhi |  |
| 176 | 1121+Pi54+Piz5-2 | IARI, New Delhi |  |
| 177 | 1121+Saltol-2 | IARI, New Delhi |  |
| 178 | 1121+xa13+Xa21-1 | IARI, New Delhi |  |
| 179 | 1121+xa13+Xa21-2 | IARI, New Delhi |  |
| 180 | 1121+Xa38-1 | IARI, New Delhi |  |
| 181 | 1121+Xa38-2 | IARI, New Delhi |  |
| 182 | 1401+Pi54+Piz5-1 | IARI, New Delhi |  |
| 183 | 1401+Pi54+Piz5-2 | IARI, New Delhi |  |
| 184 | 1401+xa13+Xa21-1 | IARI, New Delhi |  |
| 185 | 1401+xa13+Xa21-2 | IARI, New Delhi |  |
| 186 | BML-35-1 | IARI, New Delhi |  |
| 187 | BML-38 | IARI, New Delhi |  |
| 188 | PR114 | IARI, New Delhi |  |
| 189 | Rajendra Mahsuri | NRCPB, New Delhi |  |
| 190 | NKSWR63 | New collection | Ballia, Uttar Pradesh |
| 191 | NKSWR63 | New collection | Ballia, Uttar Pradesh |
| 192 | *O. rufipogon* 336687 | NBPGR, New Delhi | Balangir, Odisha |
