# Supplementary material for: Single-copy gene based 50 K SNP chip for genetic studies and molecular breeding in rice
Source: Sci Rep. 2015 Jun 26;5:11600. doi: 10.1038/srep11600 (PMC4481378; doi:10.1038/srep11600)
Supplement: Supplementary Table 4 [file srep11600-s5.doc]

**Supplementary Table 4|** Analysis of chromosome-wise percentage of background similarity with the recipient parent in submergence tolerant rice varieties based on the 50K chip data

| **1. Swarna-Sub1** | | | | | |
| --- | --- | --- | --- | --- | --- |
| Chromosome | No. of SNPs | Match | Mismatch | % Recipient | % donor |
| 1 | 10016 | 9857 | 159 | 98.41 | 1.59 |
| 2 | 6067 | 5944 | 123 | 97.97 | 2.03 |
| 3 | 7044 | 6848 | 196 | 97.22 | 2.78 |
| 4 | 3776 | 3720 | 56 | 98.52 | 1.48 |
| 5 | 4297 | 4212 | 85 | 98.02 | 1.98 |
| 6 | 5502 | 5423 | 79 | 98.56 | 1.44 |
| 7 | 2334 | 2323 | 11 | 99.53 | 0.47 |
| 8 | 2578 | 2511 | 67 | 97.40 | 2.60 |
| 9 | 2463 | 2317 | 146 | 94.07 | 5.93 |
| 10 | 1284 | 1227 | 57 | 95.56 | 4.44 |
| 11 | 2678 | 2593 | 85 | 96.83 | 3.17 |
| 12 | 2012 | 1937 | 75 | 96.27 | 3.73 |
| TOTAL | 50051 | 48912 | 1139 | 97.72 | 2.28 |

| **2. IR64-Sub1** | | | | | |
| --- | --- | --- | --- | --- | --- |
| Chromosome | No. of SNPs | Match | Mismatch | % Recipient | % donor |
| 1 | 10016 | 9467 | 549 | 94.52 | 5.48 |
| 2 | 6067 | 5516 | 551 | 90.92 | 9.08 |
| 3 | 7044 | 6362 | 682 | 90.32 | 9.68 |
| 4 | 3776 | 3578 | 198 | 94.76 | 5.24 |
| 5 | 4297 | 3929 | 368 | 91.44 | 8.56 |
| 6 | 5502 | 5165 | 337 | 93.87 | 6.13 |
| 7 | 2334 | 2108 | 226 | 90.32 | 9.68 |
| 8 | 2578 | 2368 | 210 | 91.85 | 8.15 |
| 9 | 2463 | 2119 | 344 | 86.03 | 13.97 |
| 10 | 1284 | 1097 | 187 | 85.44 | 14.56 |
| 11 | 2678 | 2400 | 278 | 89.62 | 10.38 |
| 12 | 2012 | 1795 | 217 | 89.21 | 10.79 |
| TOTAL | 50051 | 45904 | 4147 | 91.71 | 8.29 |

| **3. CR1009-Sub1** | | | | | |
| --- | --- | --- | --- | --- | --- |
| Chromosome | No. of SNPs | Match | Mismatch | % Recipient | % donor |
| 1 | 10016 | 9167 | 849 | 91.52 | 8.48 |
| 2 | 6067 | 5576 | 491 | 91.91 | 8.09 |
| 3 | 7044 | 6346 | 698 | 90.09 | 9.91 |
| 4 | 3776 | 3665 | 111 | 97.06 | 2.94 |
| 5 | 4297 | 3999 | 298 | 93.06 | 6.94 |
| 6 | 5502 | 5150 | 352 | 93.60 | 6.40 |
| 7 | 2334 | 2258 | 76 | 96.74 | 3.26 |
| 8 | 2578 | 2349 | 229 | 91.12 | 8.88 |
| 9 | 2463 | 2255 | 208 | 91.56 | 8.44 |
| 10 | 1284 | 1130 | 154 | 88.01 | 11.99 |
| 11 | 2678 | 2344 | 334 | 87.53 | 12.47 |
| 12 | 2012 | 1791 | 221 | 89.02 | 10.98 |
| TOTAL | 50051 | 46030 | 4021 | 91.97 | 8.03 |

| **4. Samba Mahsuri-Sub1** | | | | | |
| --- | --- | --- | --- | --- | --- |
| Chromosome | No. of SNPs | Match | Mismatch | % Recipient | % donor |
| 1 | 10016 | 8088 | 1928 | 80.75 | 19.25 |
| 2 | 6067 | 4661 | 1406 | 76.83 | 23.17 |
| 3 | 7044 | 5788 | 1256 | 82.17 | 17.83 |
| 4 | 3776 | 2960 | 816 | 78.39 | 21.61 |
| 5 | 4297 | 3429 | 868 | 79.80 | 20.20 |
| 6 | 5502 | 3762 | 1740 | 68.38 | 31.62 |
| 7 | 2334 | 1681 | 653 | 72.02 | 27.98 |
| 8 | 2578 | 2184 | 394 | 84.72 | 15.28 |
| 9 | 2463 | 1933 | 530 | 78.48 | 21.52 |
| 10 | 1284 | 1108 | 176 | 86.29 | 13.71 |
| 11 | 2678 | 2121 | 557 | 79.20 | 20.80 |
| 12 | 2012 | 1678 | 334 | 83.40 | 16.60 |
| TOTAL | 50051 | 39393 | 10658 | 78.71 | 21.29 |
