# Supplementary material for: Single-copy gene based 50 K SNP chip for genetic studies and molecular breeding in rice
Source: Sci Rep. 2015 Jun 26;5:11600. doi: 10.1038/srep11600 (PMC4481378; doi:10.1038/srep11600)
Supplement: Supplementary Table 6 [file srep11600-s7.doc]

**Supplementary Table 6|** List of AGCR (agronomically important cloned rice) genes with donor type alleles in Swarna-sub1 out of total 194 AGCR genes assayed in the 50K SNP chip

| **S. No.** | **LOC_ID** | **Gene copy** | **Gene name** | **Associated trait** | **Annotated function** |
| --- | --- | --- | --- | --- | --- |
|  |  |  |  |  |  |
|  | LOC_Os01g27490 | MCR | *ANS1* | Flavonoid biosynthesis | Leucoanthocyanidindioxygenase |
|  | LOC_Os01g61080 | SCR | *WRKY24* | Drought tolerance | WRKY transcription factor 24 |
|  | LOC_Os01g62020 | SCR | *UXS-4* | Seed development | UDP-glucuronic acid decarboxylase |
|  | LOC_Os04g0164900 | MCR | *SDBE* | Cooking quality | Starch de-branching enzyme |
|  | LOC_Os05g40384 | MCR | *EUI1* | Panicle elongation | Cytochrome P450 |
|  | LOC_Os06g06530 | SCR | *HGW* | Heading date and grain weight | Proline-rich cell wall protein-like |
|  | LOC_Os07g47100 | SCR | *NHX1* | Salt stress tolerance | Na+/H+ Antiporter |
|  | LOC_Os08g35510 | MCR | CYP76M10 | Bacterial and fungal resistance | Cytochrome P450,  Oxidoreductase |
|  | LOC_Os09g11450 | MCR | *NHX5* | Salt tolerance | Transport of Na+ and K+into vacuoles |
|  | LOC_Os10g41980 | SCR | *RIR1B* | Blast resistance | Rapid Alkalinization Factor RALF family protein precursor |
